# Evaluation of Monolayer and Bilayer Buccal Films Containing Metoclopramide

**DOI:** 10.3390/pharmaceutics16030354

**Published:** 2024-03-02

**Authors:** Blaž Grilc, Odon Planinšek

**Affiliations:** Department of Pharmaceutical Technology, Faculty of Pharmacy, University of Ljubljana, Aškerčeva 7, 1000 Ljubljana, Slovenia

**Keywords:** image analytics, Raman microscopy, crystallization, buccal films, bilayer

## Abstract

The objective of this study was to develop buccal film formulations containing metoclopramide hydrochloride monohydrate (MCP) with and without a backing layer and to evaluate their release properties and physiochemical stability. The crystallization of MCP in the polymer matrix was monitored with image analysis techniques for rapid and scalable observation. The results showed that the addition of a protective layer and its thickness significantly affected the release rate and crystallization behavior of MCP in the formulations. The crystallization of MCP increased over time, and certain formulations showed higher susceptibility to crystallization. To understand the factors affecting the crystallization of MCP, the relationship between the viscosity and pH of the casting solution was examined, but no significant correlation was found. A significant correlation was observed between the plasticizer concentration and the physical state of MCP. Through a systematic Design of Experiment (DOE) approach, an optimal formulation was devised, successfully preventing crystallization of the active ingredient. However, enhancing the overall chemical stability of the formulated product remains a challenge.

## 1. Introduction

The administration of drugs via the buccal route has shown benefits, primarily because it allows the drugs to bypass first-pass metabolism and improves the bioavailability of drugs that are highly susceptible to degradation after oral administration [1]. This is a promising route of administration but is unfortunately limited to only specific drugs [2]. These are usually small, potent molecules that exhibit sufficient permeability through the buccal membrane. The application of drugs to the buccal membrane is not always superior compared to oral administration, because of the lower permeability of the mucosal membrane in the oral cavity compared to intestinal mucosa [3]. This type of application might have a great advantage in the case of drugs with poor or variable bioavailability depending on patient metabolism performance [4]. Drugs that undergo extensive first-pass metabolism often exhibit varying concentrations in the blood plasma among patients [5]. The plasma concentrations of drugs play a critical role in determining both their desired therapeutic effects and undesirable side effects. Film formulations used for the buccal administration of drugs have an additional advantage over conventional tablets: they act quickly, resulting in the fast onset of drug action [6,7]. 

One of the drugs with varying bioavailability is antiemetic metoclopramide (MCP). It is usually administered to oncology patients during chemotherapy to treat nausea and prevent patients from vomiting. Many anticancer drugs have severe side effects such as nausea, so patients need supportive therapy to better withstand the main therapy [8]. MCP is available on the market as a 10 mg tablet, 1 mg/mL oral solution and 10 mg solution for injection. Its variable bioavailability was first reported by Bateman et al., who found that bioavailability in humans varies from 32 to 97% depending on the metabolic performance of the patient [9]. It was later found that the variability was more pronounced at therapeutic doses below 10 mg. Taylor et al. examined the variability of high MCP doses and found that bioavailability was more consistent when patients were given high doses of MCP (2.5 mg/kg) [10]. Nevertheless, the therapeutic doses used are within the range where variability in bioavailability was observed; therefore, the improvement of administration is a possible approach to better therapeutic outcomes.

Oral films containing MCP have been presented previously by several authors [11,12,13,14,15,16,17,18]. All the formulations described were prepared as monolayer films in which the active ingredient was homogeneously distributed in the polymer matrix. The drug MCP is freely soluble in water in a salt form and is therefore dissolved in aqueous hydrogels. The high solubility of the drug combined with the bitter taste might not be pleasant for the patient [19]. The contact time of the drug with the membrane is important for adequate absorption by the mucosa [20]. To prevent omnidirectional release of the drug and to prolong the residence time of the film, a second polymeric protective layer could be used [21]. However, the previously presented formulations lacked the additional protective and directing polymer layer. 

In sources in the literature, bilayer formulations with unidirectional release can be found [22,23,24,25]. Their advantages are as follows. Double- or bilayered formulations have a different drug release profile, compared to monolayer formulations. The backing layer prolongs the dissolution time significantly [25]. Unidirectional release formulations demonstrate improved permeability when compared to omnidirectional mucosal formulations [26]. It has also been shown that unidirectional release formulations have better permeability compared to omnidirectional mucosal formulations [27]. The protective layer also directs the release of the drug by preventing its release in the oral cavity. It reduces the exposure of the tongue to bad-tasting drugs, which could improve treatment compliance.

The crystallization of drugs from polymer films is a time-dependent phenomenon that is generally undesirable because it can alter the appearance of the formulation and its release performance. Drugs that crystalize in the formulation may form larger crystals that have a smaller surface area and dissolve more slowly compared with their amorphous analogs [28]. A certain degree of mobility is required for the crystallization of the drug molecule, which is relatively high in formulations such as buccal films [29]. The process of drug crystallization in film formulations should be monitored during the development process. Methods to determine crystallization such as DSC, X-ray diffraction, Raman spectroscopy, etc., are the most suitable but usually destructive or time-consuming. To monitor the crystallization of the film production batch in a more convenient way, a new method would be welcomed. The crystallization of a drug in film formulations might be more easily observed by comparing the visual appearance, since amorphous films are transparent and do not scatter transmitted light [30]. Crystalline film, on the other hand, is opaque and the growth of crystals is usually observed more easily. Therefore, the use of image analysis and computer vision is one way to monitor the crystal growth process [31]. Analyzing images using neural networks trained for this purpose or using a pre-trained network and applying the transfer leaning concept are viable solutions for analysis of images. This was demonstrated by Zupan et al. in the field of medicine by comparing images at different stages of bone healing in mice [32].

The objective of this study was to develop and compare buccal film formulations incorporating MCP. Additionally, these formulations were covered with a backing layer to prolong adhesion to the mucosa and achieve unidirectional release properties. Furthermore, the study aimed to prevent the crystallization of MCP within a Na alginate matrix.

## 2. Methods

### 2.1. Preparation of Films

#### 2.1.1. Monolayer Formulations

Films consisted of Na-alginate (Protanal 10/60, FMC BioPolymer, Ewing, NJ, USA), xylitol (Xylisorb DC100, Roquette, Lestrem, France) and metoclopramide hydrochloride monohydrate (Biosinth, Bratislava, Slovakia). Formulations were prepared by the solvent casting method. The polymer, plasticizer and the drug were dissolved in water and stirred with a propeller stirrer (Eurostar PWR CV, IKA-Werke, Staufen, Germany) at 1000 rpm until a homogenous solution was obtained. The casting solution pH was adjusted with 0.1 M solution of citric acid to the desired value. Entrapped bubbles were removed by sonication in an ultrasonic bath (1510E-DTH, Bransonic, Eastlake, OH, USA) for 7 min. The degassed casting solution was spread on a glass plate at a thickness of 1500 µm using a motorized coating unit (PIX 1.0, UL FFA, Ljubljana, Slovenia) with an applicator (ZHA 2000.S, Zehntner, Sissach, Switzerland). The films were dried at 60 °C for 60 min in a forced convection dryer (SP −45, Kambič, Semič, Slovenia).

The film composition and pH of the casting solution varied according to the fractional factorial experimental design. The polymer, plasticizer, MCP and pH factors were varied in two levels, which are listed in Table 1. The compositions in bold are repeated experiments at the central point of the experimental design. Factorial analysis was conducted using the Modde 13 analysis tool (Sartorius, Goettingen, Germany). The positive and negative influence of a given factor was calculated using the analysis wizard interface. Square and interaction tests were performed for each measured parameter, i.e., dissolution rate and drug crystallization. The significant factors influencing the measured parameters are presented as the results.

#### 2.1.2. Bilayer Formulations

All formulations from Table 1 were prepared in both a one-layer and two-layer configuration. For the two-layer formulations, a second protective layer was cast on top of the dried drug-containing film. The casting solution for the protective layer consisted of a 20% aqueous solution of hydroxypropyl cellulose (Klucel ELF, Ashland, Covington, KY, USA). The solution was prepared by dispersing the polymer in water heated to 70 °C. While the dispersion was cooled to room temperature, it was continuously mixed with a propeller stirrer. The cooled solution was spread at a thickness of 500 or 1000 µm. The applicator setting for the second layer was adjusted based on the thickness of the first layer to ensure a uniform spreading height of the protective layer. The second layer was then dried at 40 °C for 60 min. After drying, the films were cut into dimensions of 20 × 30 mm and stored individually in airtight primary packaging to preserve their integrity. Bilayered formulations were labeled with an additional letter, “D” or “E”, depending on the thickness (70 or 110 µm) of the second layer.

Formulations with different thicknesses of the second layer were prepared in order to pre-test their influence on the release rate of the drug. An M8 formulation was used as the first layer onto which the HPC layer was casted at 500, 1000 and 1500 um. The dried thicknesses of the HPC layers were 70, 110 and 240 µm, respectively.

### 2.2. Release Rate

#### 2.2.1. USPI

Film dissolution tests were performed using a USP I (Vankel Varian VK 7000, Agilent, Cary, NC, USA) apparatus with the basket method. A volume of 500 mL was used for as the release medium. The release medium was 0.1 M phosphate-buffered solution with pH 6.8. To ensure accurate results, the films were placed vertically into the basket and mounted on the rotating shafts just before the start of the test. This precaution was taken to prevent the films from absorbing moisture prior to the release test, which could affect MCP release rate. The test was performed at a rotation speed of 50 rpm to provide consistent agitation and promote the release of the MCP from the films. Samples were taken at specific time intervals: 2, 6, 10, 15, 20, 25, 40, and 60 min. Timepoints of 80 and 100 min were added for “E” formulations. For sample collection, 10 mL of the dissolution medium was withdrawn and then filtered using 0.45 µm RC membrane filters to remove undissolved particles. The first five milliliters of the filtered sample was discarded as waste to ensure that the collected sample was representative of the dissolved components. To maintain a constant volume, the withdrawn sample volume was replenished with fresh buffer solution.

#### 2.2.2. Innovative Cell for Film Release 

Our research team has developed an innovative cell for release of films evaluation (ICRF), which was introduced to the public in 2020 and described in detail in publication [33]. The ICFR system consists of a dual-chamber flow cell divided by a membrane. This flow cell has two inlets and one outlet. During evaluation, a film is positioned on the membrane and held securely in place by the edges of the cell chamber, ensuring continuous contact with the membrane throughout the measurement process. A cellulose acetate membrane with 0.45 µm pores (Sartorius, Germany) is used as the support. The ICFR system exerts a constant hydrostatic pressure in both chambers of the flow cell. The laminar fluid flow design and chamber configuration naturally prolongs dissolution times compared to other methods. This feature proves to be advantageous as it allows for the detection of minor differences between formulations. To establish a pressure gradient, the pressure in the donor chamber was set 490 Pa higher than in the acceptor chamber. The medium flows through the acceptor chamber at a rate of 20 mL/min. Sampling is performed at four-minute intervals using an autosampler, which ensures the consistent and accurate collection of samples. The inlet medium is preheated to 37 °C and the temperature in the flow cell is recorded at one-second intervals, allowing detailed temperature monitoring throughout the experiment. The dissolution profiles were compared with the model-independent approach in accordance with international guidelines by calculating the similarity factor f2. The 90% confidence interval for f2 was calculated by bootstrapping (*n* = 5000). Dissolution profiles were accepted as similar if the lower confidence interval of f2 was greater than 50. Calculations were performed using the Excel add-in DD Solver 1.0.

### 2.3. Raman Mapping of Film Surface

The films were analyzed by Raman microscopy. For this purpose, an Xplora Plus (Horiba, Loos, France) SAS Raman system coupled to an Olympus BX43 (Olympus, Tokyo, Japan) microscope was used in combination with LabSpec 6 software (Horiba, Kyoto, Japan). Raman microscopy mapping was used to investigate the crystal state of the drug and its position in the film. Mapping was performed at 20× magnification. The measurement points were 30 µm apart, resulting in 1230 signal acquisition points. The spectra were recorded from 30 to 2000 cm^−1^ with an acquisition time of 15 s and three repetitions. The obtained spectra were further preprocessed using three methods. First, a rubber band baseline correction was applied. Second, the spectra were denoised using a Savitzky–Golay filter with a window setting of seven adjacent measurements and a polynomial order of three. Third, the spectra were normalized using vector normalization. The peak shifts at 1320 and 1530 cm^−1^ were followed to determine the physical state of the MCP.

Mapping of the film cross section was performed at 100× magnification with measurement points spaced 2 µm apart. Signal acquisition lasted 40 s with three repetitions. The peak height at 741 cm^−1^ was compared to determine the amount of the drug present in the film cross section. To prepare the sample for analysis, the film was immersed in liquid nitrogen until the boiling of the nitrogen ceased, and then the film was bent until it broke. The sample was fixed between two glass plates to expose its cross section to the microscope.

### 2.4. Image Analytics of Formulations

Each film batch was scanned using an optical scanner (Perfection V700 Photo J221A, Epson, Nagano, Japan) with a resolution of 1200 dpi and 24-bit color depth. The images were further processed to extract the individual frames of the films. The film object covered more than 90% of the total image area. The images were embedded into the Inception v3 neural network (Alphabet, Mountain View, CA, USA). The retrieved data were analyzed using Orange v3.35 data mining software (UL FRI, Ljubljana, Slovenia). A logistic regression model was developed to distinguish between dissolved and crystalized MCP on the film surface. A set of 52 manually classified images of films was selected for the development of the classification model. Logistic regression was regularized by Lasso regularization at a cost strength of 500. The model was trained in 50 iterations by random sampling from the training data at a size of 50% of the reference image set.

### 2.5. Preparation of Drug in Amorphous State

MCP was prepared in its amorphous state by differential scanning calorimetry (DSC). Initially, 5 mg of MCP was carefully weighed into a 40 µL alumina DSC pan. The sample was then placed in the DSC measurement cell (DSC1 Mettler Toledo, Greifensee, Switzerland). A heating program ranging from 0 to 200 °C was performed at a heating rate of 10 K/min. A DSC curve was observed throughout the heating process. When the sample reached its melting point (186 °C), the cover of the measurement cell was removed and the pan containing the melted MCP was quickly immersed in liquid nitrogen. The sample was then transferred to a desiccator with zero percent relative humidity and left for 30 min to ensure that any residual gas was completely removed. The so-prepared MCP sample state was then subjected to Raman microscopy analysis to obtain the reference spectra. After the acquisition of the spectra, the amorphous state of the MCP was re-evaluated by subjecting the sample to a repeated heating program in DSC. This process served to confirm the stability of the amorphous MCP. A similar study on the solid state of MCP was previously performed by Wang et al. [34].

### 2.6. Film Thickness

The film thickness was determined using a micrometer gauge model ID-U1025 (Mitutoyo, Kanagawa, Japan). To ensure accurate measurements, the entire surface of the measuring tip was covered with the film surface area during each measurement. This approach helped to minimize potential distortions in thickness readings that could occur at the edges of the film. The film thickness was measured with an accuracy of 0.01 mm. To calculate the thickness of the protective layer, the thickness of the drug film was subtracted from the overall double-layer thickness.

### 2.7. Stability

A double-sided aluminum pouch (Triplex PET12/ALU12/PE75, LogaPak, Logatec, Slovenia) was utilized as the primary packaging for the films. Each film was sealed within the pouch to prevent moisture ingress or egress. The packaging also effectively blocked light exposure. To assess the stability of the films under accelerated conditions, a stability study was conducted at 40 °C and 75% relative humidity for a duration of 90 days. Four time points were selected for evaluation: 0, 30, 60 and 90 days. On day 0, the test of five formulations was analyzed, while four films were evaluated for each subsequent time point. In addition to monitoring chemical stability, the progression of the crystallization of MCP at each time point was monitored using image analysis techniques. This allowed for the evaluation of any changes in the extent of crystallization over the course of the stability study.

### 2.8. Viscosity of Casting Solution

The viscosity of the casting solutions was determined with a rotational viscometer (M301, Anton Parr, Graz, Austria) using the cone and plate method. The PP50 spindle (Anton Paar, Austria) was used, positioned on a flat surface at a distance of 1 mm. To ensure consistency, the measuring cell was cooled to 20 °C during the measurements. Measurements were performed by varying the shear rate from 1 to 100/s. By systematically increasing the shear rate, it was possible to characterize the flow behavior of the casting solutions over a range of shear conditions.

### 2.9. Metoclopramide Assay

#### 2.9.1. HPLC

The chemical stability of the MCP was evaluated using the HPLC method outlined in the European Pharmacopoeia 11th edition, monograph 04/2018:0674 [35]. The HPLC system utilized for the analysis was Agilent1260 (Agilent, Cary, NC, USA), which consisted of the G7115A DAD detector, Phenomenex Gemini C18 110A column, 3 µm, 150 × 4.6 mm, G7167A multisampler and the G7112B binary pump. The dedicated software for the data analysis was OpenLAB CDS Version 2.3 by Agilent (USA).

Mobile phase A: 5 g/L solution of ammonium acetate (Merck, Darmstadt, Germany) in water adjusted to pH 5.0 with acetic acid (Merck, Germany).

Mobile phase B: Acetonitrile (J.T. Beaker, Radnor, PA, USA).

Gradient elution according to Table 2 was used at a flow rate of 0.9 mL/min. Sample injection volume was 5 µL. The detection was recorded at 275 nm with a slit range of 4 nm.

The stability performance of the method was assessed by preparing samples containing MCP degradation products. They were prepared from a solution of MCP at a concentration of 0.1 g/L that was exposed to a UV-C light source with a rated power of 30 W for 5, 15 and 30 min. Additionally, an oxidative method using 6% hydrogen peroxide (Merck, Germany) was employed to generate degradation products. A 200 µL of 30% hydrogen peroxide was transferred in the HPLC vial containing 800 µL of 0.1 g/L MCP solution. The vial was closed and agitated. The sample was injected into the chromatographic system after one, three and 24 h. The vial with the sample was kept at 20 °C during injections. 

To determine the MCP content in the films, one film was dissolved in a 100 mL volumetric flask with phosphate buffer at pH 6.8. The resulting solution was mixed at 500 rpm for 60 min using an IKA RO15 magnetic stirrer (IKA, Germany). Then, 2.0 mL of the solution was transferred to a test tube and diluted with 3.0 mL of acetonitrile. After 20 min, the solution was filtered through a 0.45 µm RC membrane filter. The first 4 mL of the solution was discarded, and the remaining solution was collected in an HPLC vial. Acetonitrile dilution was used to precipitate the Na-alginate to prevent clogging of the HPLC system. The recovery of MCP during the precipitation of Na-alginate was studied by preparing placebo formulations spiked with a known amount of MCP.

#### 2.9.2. UV-VIS

The concentration of MCP during the dissolution test was determined using a Carry 60 UV-Vis spectrometer (Agilent, USA). Measurements were performed in a quartz cuvette with an optical path length of 10 mm, at a wavelength of 275 nm. A calibration curve was constructed for the concentration range of 0.005 to 0.05 g/L. The equation of the calibration curve for MCP was determined as 34.196 × A − 0.006, which gave an R^2^ value of 0.9999. The linear response was observed in the absorbance range of 0.05 to 1.78. To ensure accuracy, the background spectra of all excipients used were examined and found to have no peaks at 275 nm, even at twice the expected concentrations.

## 3. Results and Discussion

### 3.1. Visual Observation of the Obtained Films 

#### 3.1.1. Monolayer Films

**Figure 1:**
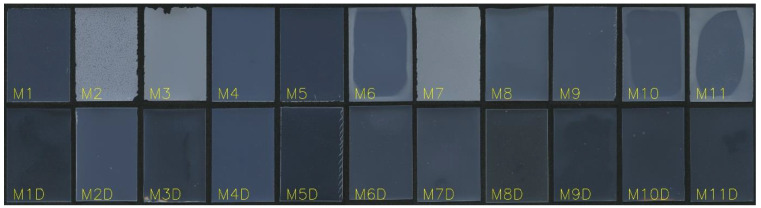
One representative film of each formulation at day 0.

The experimental plan outlined in Table 1 was followed to prepare the films. Upon initial visual observation, the film properties were assessed. It was observed that none of the film casts were broken after drying and very few trapped bubbles were present. However, formulations M2, M3 and M7 and the central point formulations (M6, M10 and M11) exhibited non-transparency and had visible fine particles on the film surface (Figure 1). This was attributed to the crystallization of MCP. These formulations contained a low level of plasticizer xylitol. It seems that the solubilizing capacity of the film matrix was exceeded in these formulations. Films M2 and M3 also had broken edges, indicating poor cutting properties and potentially low mechanical strength. Despite the broken edges, these formulations could still be handled without further breakage.

Formulations M1 and M8 demonstrated optimal visual properties, with a homogeneous film surface, the absence of visible distortions, and well-defined edges. These formulations contained a low level of MCP and high level of Na-alginate in the casting solution. 

#### 3.1.2. Bilayer Films

The bilayer films exhibited transparency without the appearance of crystalized MCP. Even films prepared with a high level of MCP, such as M2D and M7D, remained transparent without visible particles after drying. Although white opaque crystals were present in the first layer, they dissolved when the second layer was applied and remained dissolved after the drying process. This led to the assumption that crystals in the monolayer films formed mainly on the top (air-facing) surface, since no crystals were observed on the bottom side. This can be explained by moisture diffusion during evaporation, where a moisture concentration gradient forms within the film [36]. As the film surface becomes less moist, MCP becomes supersaturated, which eases crystallization. The application of the second layer, which contained a water solution of HPC, dissolved the MCP crystals that had formed on the surface and the film became transparent. The transparency was maintained even after drying.

### 3.2. MCP Release Comparison of Mono- and Bilayer Films by Two Dissolution Methods

**Figure 2:**
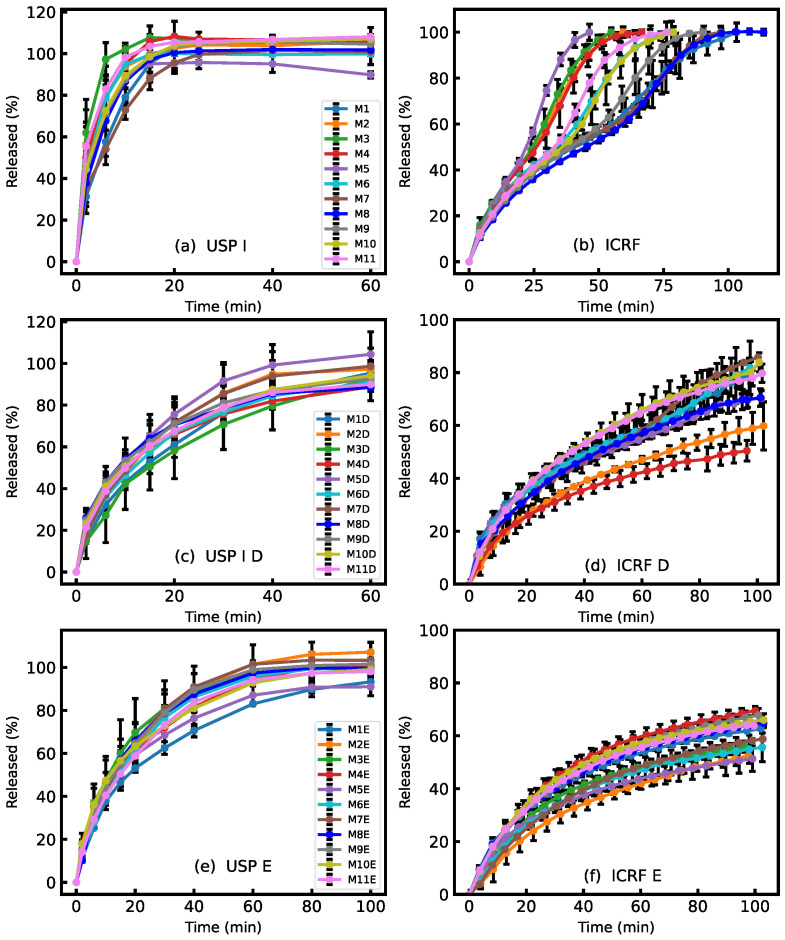
Comparison of MCP release from monolayer and bilayer formulations: (**a**) monolayer formulations USP I; (**b**) monolayer formulations ICRF; (**c**) bilayer formulations USP I 70 μm; (**d**) bilayer formulations ICRF 70 μm; (**e**) bilayer formulations USP I 110 μm; (**f**) bilayer formulations ICRF 110 μm.

The release rate of MCP from films was evaluated using the USP I dissolution system and the ICRF method. Both methods are recognized as able to distinguish differences in the release rates of buccal films [33,37]. The main aim of the dissolution tests was to observe the effect of the backing layer on the release rate of the films. It was assumed that adding an additional layer to the formulation should affect the release rate of the films, but the order of release and the relations between profiles should remain similar to that of the monolayer films. The HPC polymer used in the second layer is a water-soluble polymer and its release rate is lower than that of Na-alginate [38]. The HPC polymer was selected because it dissolves more slowly than the main polymer, acts sufficiently as a barrier and can dissolve completely in the mouth. Therefore, no film residue needs to be removed from the oral cavity after application. However, the assumption that this would have little or no effect on the release sequence has been found to be incorrect. The dissolution profiles shown in Figure 2 indicate that the release profiles of films with a protective layer is slower compared to films without one. The slower release rate of bilayer films was already demonstrated by several authors [39,40]. The ranking of the films within the same methods changed, indicating that the protective layer does not affect all formulations equally, despite the composition of the MCP layer remaining the same.

**Figure 3:**
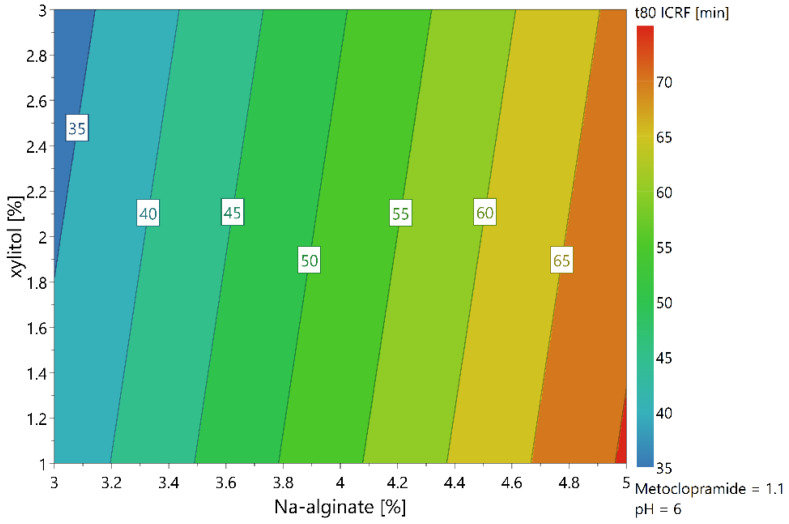
Contour plot of predicted 80% release time in dependence of concentration of Na alginate and xylitol at 1.1% of MCP and pH 6 for monolayer formulations.

Factorial analysis of the experimental design revealed that the amount of Na-alginate was the main factor affecting the release rate of the monolayer films, as observed in both the USP I and ICRF methods. The release testing results were modeled using the statistical tool Modde 13. Figure 3 shows the contour plot of predicted release times for the ICRF method. The obtained statistical model parameter R^2^ was 0.97, and the reproducibility factor was 0.96, indicating that the model had a high agreement with the experimental data. The prediction suggests that the dissolution time can be extended by increasing the concentration of Na-alginate and decreasing the concentration of xylitol in the casting solution. The order of release profiles is not identical for the USP I and ICRF methods. In the ICRF method, there is a greater difference between the formulations at 80% release, indicating that the ICRF method can better discriminate differences between formulations compared to USP I. The profiles of repeated central point experiments M6, M10 and M11 were comparable; their similarity factor f2 was 74.8 when treating M6 as a reference. The average variability of the USP I method was 4.8%, and of the ICRF method, 2.6%. The trend of release profiles between the two methods was similar, but the overall variability was greater in the USP I method. Therefore, we conclude that higher repeatability can be achieved by using the ICRF method for the release testing of such formulations. 

**Figure 4:**
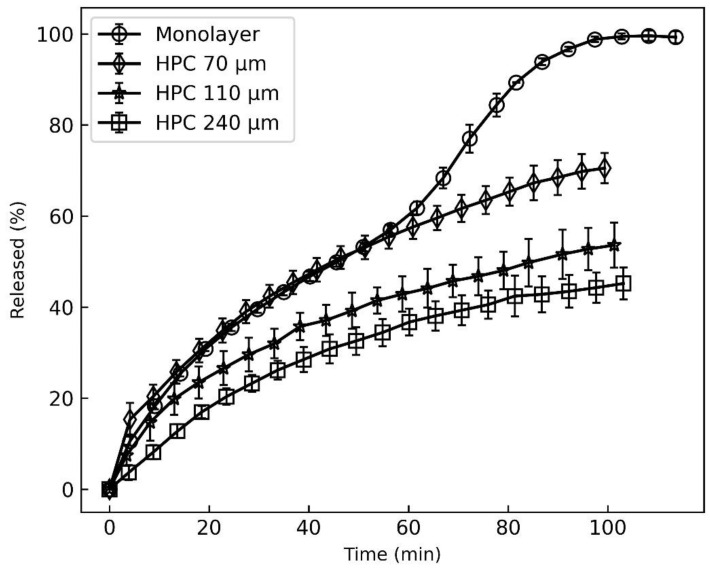
Release profiles of bilayer films with different thicknesses of HPC layer.

The bilayer formulations’ release ranking order (Figure 2c–f) was not correlated with any of the studied factors from the experimental design, i.e., polymer, plasticizer, metoclopramide amount and pH. However, the application of the second layer significantly affected the drug release rate. Based on the MCP release characteristics of the monolayer formulations, it is difficult to predict its release from bilayer films. When developing multilayer formulations, it is important to test the film’s properties after both layers have been added, even if the second layer composition is not modified. To investigate the causes of unexpected release rates from bilayer formulations, additional tests were performed. The thickness of the second HPC layer was varied by the casting applicator settings, which were 500, 1000 and 1500 µm, respectively. The drug layer thickness and composition were kept unchanged in all formulations, and the composition of M8 film was used as the drug layer. The prepared films were tested using the ICRF method. The results shown in Figure 4 demonstrate that the release order of MCP from bilayer films is affected by the thickness of the HPC layer. The significant differences between the release rate profiles with an HPC layer thickness of 70 and 110 µm were found by calculating the similarity factor f2, which was 46.4. The similarity factor of HPC 110 µm and HPC 240 µm release profiles was 59.1. This suggests that films with a thicker backing layer had similar release. However, the release rate decrease is not linearly proportional to the thickness of the HPC film. All bilayer formulations in the experimental design were prepared with an HPC layer thickness of 70 µm. This might explain the observed release ranking change of the bilayer formulations compared to monolayer formulations. Since the HPC film is relatively thin, it is particularly susceptible to variations in its release behavior. By increasing the thickness of the HPC layer, it might be possible to reduce the variability of the film release rates.

To test this hypothesis, another batch of bilayer films was prepared with a thicker backing layer. These formulations were labeled with letter “E”. A setting of 1000 μm was used to prepare a backing layer with a thickness of 110 μm. The release profiles of these formulations were then measured. From the results (Figure 2e,f), it was observed that the release rate of E films was slower compared to D films. The overall variability in the dissolution profiles decreased. With the USP I method, the average standard deviation decreased from 5.9% to 4.6%. In the ICRF method, a decrease in standard deviation was also observed, from 3.4% for D formulations to 2.2% for E formulations. The results show that the thickness of the backing layer reduces the variability of the drug release rate. 

### 3.3. The Analysis of Drug Crystallization in the Film by Raman Microscopy

**Figure 5:**
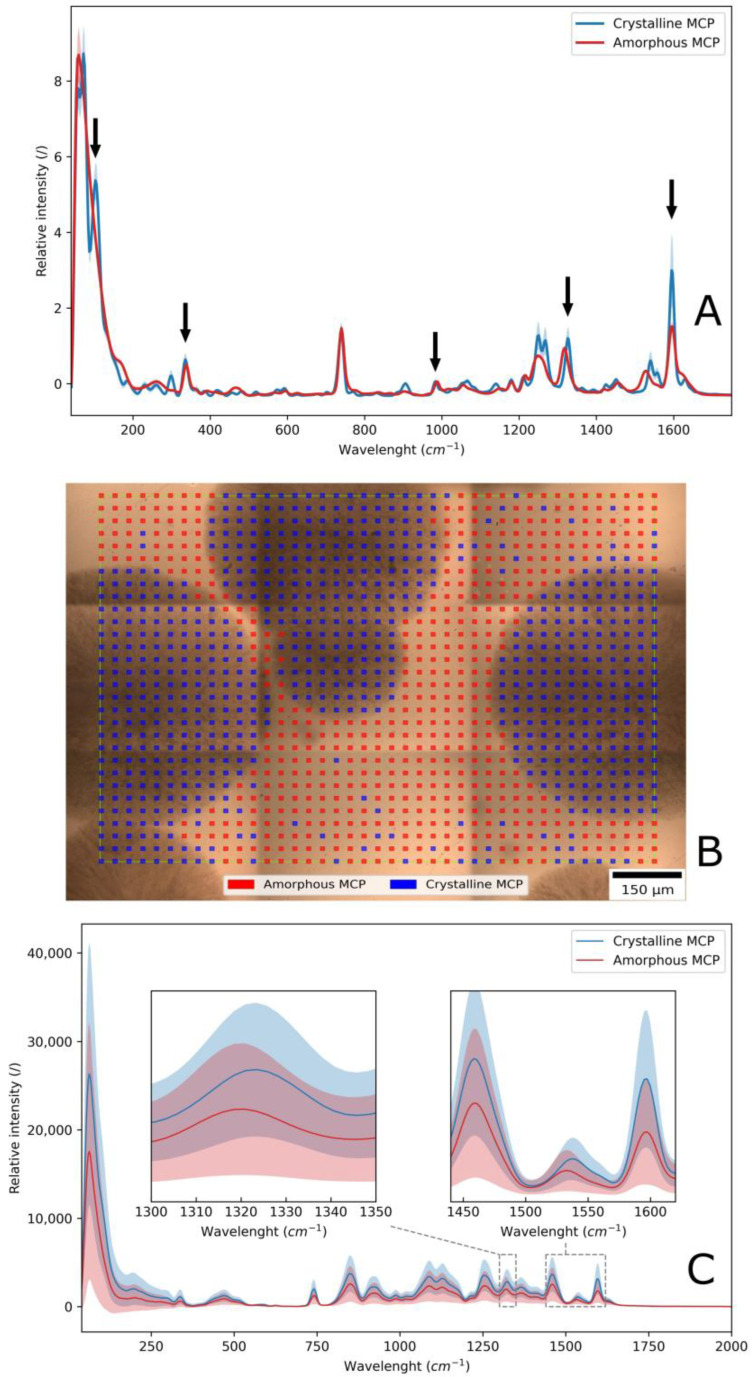
(**A**) Differences in average Raman spectra between crystalline and amorphous MCP; arrows point to the peaks where Raman shift was observed. (**B**) Raman mapping of a surface that contained opaque and transparent regions in the film. (**C**) Average Raman mapping spectra and their standard deviation, with the regions of interest magnified.

The drug MCP is highly soluble in water and also soluble in the dried film matrix. Na-alginate and xylitol can stabilize MCP and prevent its crystallization. The composition of the films was chosen to be close to the solubilizing capacity of the film matrix to study the crystallization of MCP in the films after drying. The DSC method was initially used to determine the crystal state of the drug in the film. Unfortunately, it did not provide relevant results because the amount of MCP in the film was less than 20%. Also, the enthalpy changes of the excipients increased the background noise, so potential melting peaks of MCP were not detected. Raman microscopy was the method that enabled the evaluation of the drug physical state in the film. First, all raw components were evaluated by Raman microscopy to obtain reference spectra for each ingredient. Then, the Raman spectra for crystalline and amorphous MCP were obtained. The differences between the Raman spectra of amorphous and crystalline MCP are shown in Figure 5A. The average spectra were obtained by the acquisition of 20 amorphous and 39 crystalline spectra. It is observed that crystalline spectra contain more peaks than the amorphous samples. Most prominent spectra differences were observed at 103 cm^−1^, where the amorphous MCP does not contain a peak. Raman shifts of crystalline peaks were observed at 336, 740, 983, 1326 and 1595 cm^−1^. The identified peaks were already studied in more detail by Leopold et al. [41]. The peaks in the amorphous MCP shifted to 339, 742, 988, 1317 and 1597 cm^−1^, respectively. Furthermore, differences in the shapes of the spectra were observed in the ranges of 40–80, 1200–1300 and 1500–1550 cm^−1^. 

After identifying the differences in the Raman spectra of amorphous and crystalline MCP, a mapping of films was performed. A film sample with opaque and transparent areas was mapped by Raman microscopy. The Raman mapping results are shown in Figure 5B, in which the color map represents the location of dissolved MCP (red overlay) and the MCP in the crystal state (blue overlay). Crystals formed on the surface of the film; this was observed by inspecting the films under a microscope at different focal point depths. Crystals had a gray and more opaque appearance. The averaged mapping Raman spectra of the film are presented in Figure 5C. The polymorphic state of the drug was explored by an examination of the peak shifts at 1320 and 1530 cm^−1^. The peaks at these wavelengths exhibited the greatest shifts, so they were selected for a crystallization study. The amorphous state of MCP is found in the transparent areas of the film, and the crystalline form is found in the opaque areas. The study revealed that the MCP state can be distinguished by observing the film’s visual appearance. The visually observed results comply with the measured Raman spectra. 

### 3.4. Analysis of MCP Physical Stability by Image Analytics

**Figure 6:**
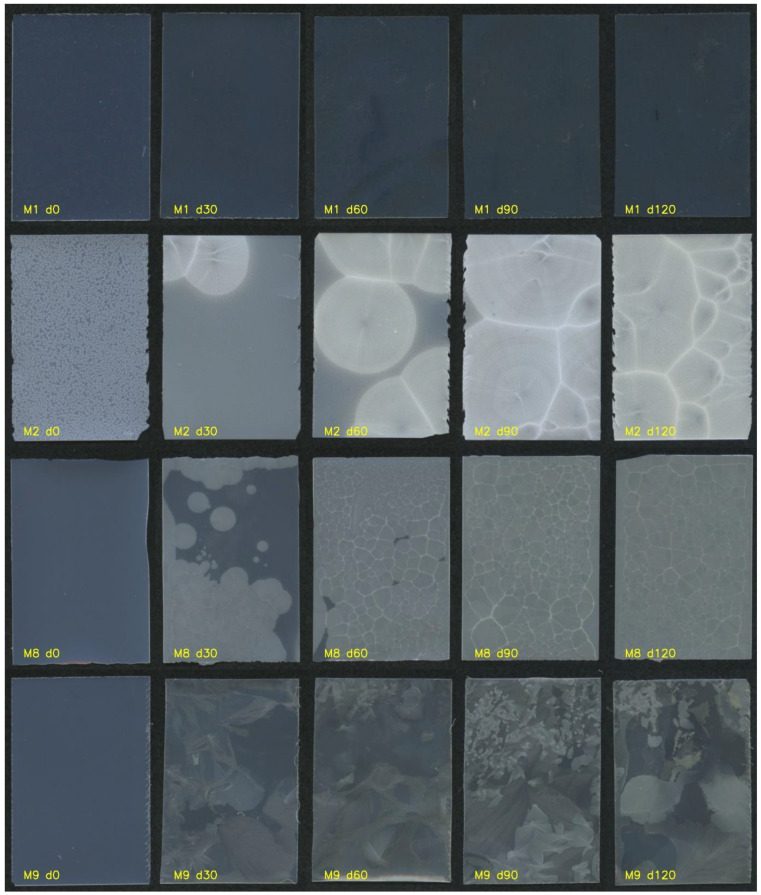
Crystallization of MCP through time in different formulations.

Visual observation of films was paralleled with Raman mapping for the examination of the physical state of the MCP and its stability monitoring. Computerized image analysis was introduced to allow larger amounts of film to be examined more quickly yet reliably. A 4-month accelerated stability study was performed under conditions of 40 °C and 75% relative humidity. The five stability time points were 0, 30, 60, 90 and 120 days. At each time point, the films were unwrapped, and their image was captured with an optical scanner. The images were embedded into an Inception v3 neural network. The retrieved data were evaluated and classified into two clusters, “crystalline” or “amorphous”. The reference pool of images was created from 52 images, 34 crystalline and 18 amorphous. These data were used as the training set for the logistic regression model. The classification accuracy of the model was tested by 50-time repeated random sampling of the training data set at 50%. The model classification accuracy was 94%. A total of 452 individual images of film formulations were classified by the obtained logistic regression model. Each formulation dataset consisted of five samples at day zero and four samples at later time points. Figure 6 shows the crystallization of MCP in the different formulations over the duration of the stability study. From images, it is observed that crystallization was an ongoing process as the size of the crystals increased through time. The shape of the crystals that formed was not the same in all formulations. For example, formulations M8 and M2 had concentrically shaped crystals with a well-defined boundary between the crystallization centers. In contrast, the shape of the crystals in formulation M9 was different, and an increase in crystallization over time was still observed. A similar shape of crystals as M9 was also observed in formulation M4. Both formulations contained a higher amount of xylitol compared to M2 and M8, which could be the reason for the change in the shape of the MCP crystals.

**Figure 7:**
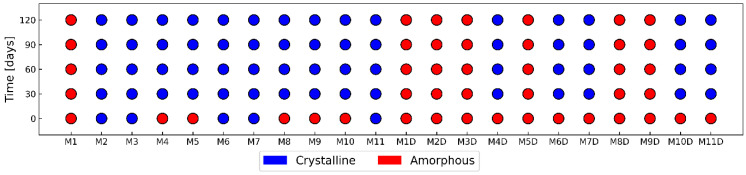
MCP physical state in freshly prepared and aged monolayer and bilayer (D) films, based on analysis of images.

The results of the classification of the images are shown in Figure 7, which shows the crystallization map of MCP over the time of the stability study. All samples within the same time point were placed in the same group. However, at 13% of the sampling points, the classification was not consistent for all samples within the group, so the classification was equal to the major decision of the classification model. On day zero, when the films were freshly prepared, a higher percentage of amorphous formulations was observed. Only five monolayer formulations contained MCP in crystalline form: M2, M6, M7, M10 and M11. Formulations M2 and M7 contained a higher concentration of MCP in combination with a low amount of plasticizer, resulting in the crystallization of MCP already in the freshly prepared films. Formulation M3 was prepared with low amounts of Na alginate (3%) and plasticizer (1%) and was also crystalline on the day of preparation despite the low MCP concentration. Formulations M6, M10 and M11 were the central point experiments and contained the same composition. M10 was recognized as amorphous at day of preparation, because the 60% of samples in its group were classified as amorphous. 

Based on the image analysis results, the most important factors affecting crystallization were identified. Table 3 shows the factors and their significance in the crystallization of MCP. Increasing the amount of MCP in the formulation promoted crystallization, which was expected. Increasing the amounts of Na-alginate and xylitol at higher pH was preventing crystallization. The amount of xylitol had the greatest significance, Na-alginate was found to be the second most important factor, and the pH of the casting solution was the least important. The crystallization of MCP was sufficiently prevented only when Na-alginate and xylitol were increased together rather than individually. This is shown by film from formulation M5, which contained xylitol at a high level (3%), and M8, which contained a high level (5%) of Na alginate in the casting solution. Both formulations contained the same level of MCP (0.8%) as M1 in the casting solution, and only in M1 did MCP not crystalize, because it contained high levels of both xylitol and Na-alginate.

After 30 days, the crystallization of MCP was induced in the majority of monolayer films. M1 was the only monolayer formulation that retained MCP in an amorphous state, even after 120 days of the accelerated stability study. The crystal map remained unchanged from 30 to 120 days of the stability study.

None of the bilayer formulations contained crystalline MCP on the day of preparation, and less than half formulations contained crystals after the 30-day timepoint. Crystallization was observed in formulations M4D, M6D, M7D, M10D and M11D. Formulations M4D and M7D contained high levels of MCP, so crystallization was expected. Formulations M6D, M10D and M11D had all the same composition; they were replicates in the central point of the experimental design, so crystallization occurred equally. Crystallization was sufficiently inhibited also in the formulations M2D, M3D, M5D, M8D and M9D.

**Figure 8:**
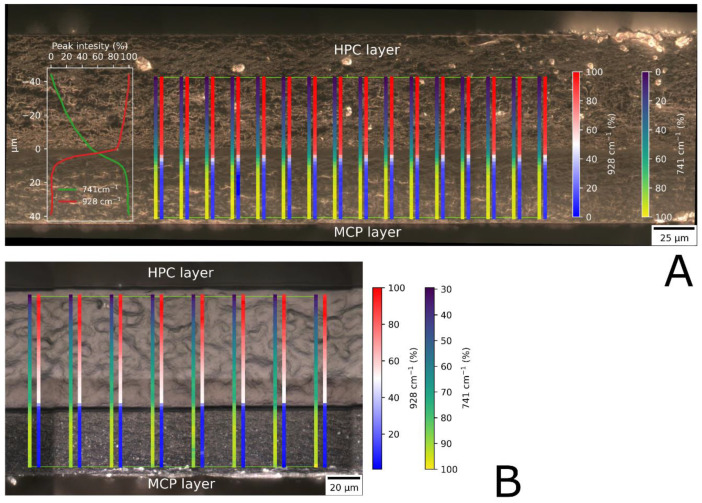
(**A**) Peak intensity of MCP and HPC at the cross section of a bilayer film M11D as determined by Raman mapping. (**B**) Peak intensity of MCP and HPC at the cross section of a bilayer film M2D as determined by Raman mapping.

During the preparation of bilayer films, an interesting phenomenon was observed. Monolayer films that contained crystals were non-transparently opaque in appearance. Immediately after the application of the second layer, the film became transparent because the white crystals of MCP dissolved. It was assumed that the crystals formed only on the surface of the main film. After the second layers of the films were dried, their appearance remained transparent, which suggests that the MCP remained in the dissolved state. We suspect that dissolved MCP could also diffuse into the backing layer and, therefore, its concentration at the junction between films decreases, which prevents precipitation. To confirm this hypothesis, the MCP concentration at the interface between the layers was investigated by cross-sectional analysis of the films using Raman microscopy. The results of Raman mapping are presented in Figure 8A. Raman mapping was performed perpendicular to the boundary between the two layers, and the results are shown as a color map. The Raman peak at 741 cm^−1^ is a characteristic peak of MCP. Other excipients do not show a peak at this wavelength. At 928 cm^−1^ is a characteristic peak of HPC, which is unique compared to other film components. The intensity of the peak corresponds to the concentration of the measured compound. The visual boundary between the two layers is clearly visible. The color represents the relative signal intensity of a peak in percent compared to the maximum signal height at the measured wavelength. The mini graph on the left side represents the average of all 16 vertical maps. The concentration gradient in the MCP layer is observed to reach a depth of 15 µm, which corresponds to 38% of the total MCP layer thickness. The obtained results indicate a decrease in MCP concentration at the junction of the two layers. At the same time, the diffusion of MCP from the main film into the HPC layer is observed. The mapping also revealed the concentration gradient of the HPC polymer in the backing layer is reversely proportional to the MCP gradient. Similar results were also obtained in other tested bilayer formulations (Figure 8B). The results suggest that after the application of the HPC layer, the drug layer is locally diluted at the junction of the two films, which prevents the crystallization of MCP in the bilayer formulations during drying and after storage.

No correlation was observed between the crystal state of MCP and the order of release of the films. It is well known that amorphous materials exhibit a faster dissolving rate than crystalline [28]. However, the release rate of MCP in presented formulations was presumably limited by the dissolution rate of Na-alginate. The latter was slower compared to the release rate of amorphous or crystalline MCP. Therefore, the influence of MCP’s crystal state was not observed during the release. From the results, it was concluded that the M1 formulation performed the best in terms of the physical stability of MCP by retaining its amorphous state. 

### 3.5. Viscosity of the Casting Solution

**Figure 9:**
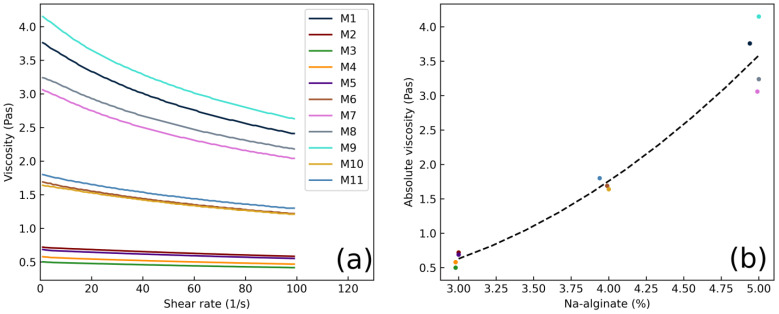
(**a**) Rheograms of casting solutions M1–M11; (**b**) absolute viscosity in dependence on concentration of Na-alginate in the casting solution.

The casting solution viscosity of all formulations was measured. The aim was to investigate the possible relationship between the viscosity of the casting solution and the crystallization of MCP. The rheograms of formulations M1–11 are shown in Figure 9a. The three viscosity classes of the casting solutions were measured. The viscosities are related to the concentration of polymer in the casting solution, which was the main factor increasing the viscosity. As observed from Figure 9b, the absolute viscosity of the casting solution is proportional to the increasing amount of Na-alginate. A deviation from the linear increase was most likely due to the xylitol presence in the casting solution. Formulations M1 and M9 contained a high amount of xylitol, and their casting solution viscosity was higher compared to M8 and M7. Although the casting solution viscosity correlated well with the amount of Na alginate, no correlation was observed between the casting solution viscosity and the crystallization of MCP during film drying. This suggests that Na-alginate, which primarily affects viscosity, is not the main factor in inhibiting MCP crystallization. This further supports the hypothesis observed from the factorial analysis that the amount of xylitol in the formulation is the main factor affecting the crystallization of the drug. 

### 3.6. Chemical Stability of the MCP Film Formulations

**Figure 10:**
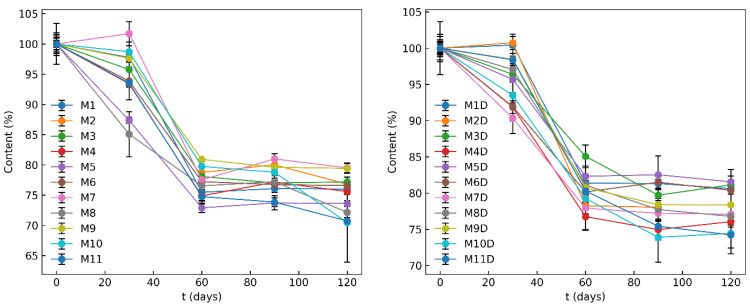
Chemical stability of monolayer and bilayer formulations containing MCP.

The results of the accelerated stability study (40 °C, 75% RH) on monolayer and bilayer formulations are depicted in Figure 10. They show a non-linear degradation profile for MCP, with a significant decrease in MCP content observed between the second and third time points in both formulation types. After 60 days, the rate of MCP degradation slows down, indicating that major degradation of MCP occurs within 60 days or less. Due to an unexpected decrease in MCP content at the 30-day mark, the study was extended to 120 days.

In general, bilayer formulations exhibited slightly better stability, with an average MCP content of 78% after 120 days, compared to monolayer formulations, which were found to contain 75% after 120 days. The amount of MCP in the formulations was found to affect stability, with higher MCP concentrations showing less degradation compared to lower concentrations. However, no correlation was found between the formulation stability sequence and the studied factors, including the amount of the drug in the film, Na-alginate, xylitol and pH. Crystallization could be beneficial for increasing stability, as the crystallized compounds have lower free energy and are generally more stable than their amorphous analogs [42]. The crystallization map indicated that certain formulations did not undergo crystallization, which might lead one to assume lower stability of MCP. However, the stability study did not observe a direct correlation between the crystal state of MCP and the stability differences between formulations. This suggests that factors other than crystallization and the quantitative ratio of components are influencing the stability. Based on the shape of the degradation profiles, it is hypothesized that there is a component involved in the degradation reaction that was not considered, such as the presence of air or moisture. This component may have played a role in the degradation of MCP until it was depleted, after which the degradation rate of MCP ceased. A similar conclusion was also made by a team of researchers developing transdermal patches with MCP [43]. According to Shan-Yang, the molecular structure of MCP has a phenyl ring and various polar functional groups, which can interact with other excipients. In particular, three structural elements, an aromatic moiety, a carbonyl group and an amide and/or tertiary nitrogen, can easily interact with the receptor sites of materials [44]. Further investigation is necessary to explore the specific mechanism of MCP instability in these formulations. Consequently, changes in the formulations or production process might be introduced to prolong the product shelf life, which is currently estimated to be 1.5 months at 40 °C. 

## 4. Conclusions

This research investigated various aspects related to the formulation and evaluation of MCP buccal films. The evaluation of MCP crystallization has been objectively followed by image analysis. This approach offers the advantage of fast analysis and the ability to cover a larger number of samples and improving the efficiency of the evaluation process. The application of a second, protective layer has a non-negligible effect on the properties of the final MCP formulation. The concentration gradient created by this additional layer during its preparation significantly affects the release profiles of MCP. This effect should be carefully considered during formulation development. The crystallization of MCP was influenced by the amount of xylitol and Na-alginate, while the viscosity of the casting solution had no significant effect. A correlation between the release rate and the crystal state of the drug was not observed because the polymer dissolution was the release control component. The application of a second layer was an effective strategy to prevent the crystallization of MCP in the final product. The chemical degradation mechanism of MCP could not be conclusively determined from the available data, indicating the need for its further investigation. The stability of the active substance in the studied films is relatively short and most likely requires the addition of appropriate stabilizers.

## Figures and Tables

**Table 1:** Composition of formulations M1–M11 in mass percentage; bold—central point experiments.

Formulation	Metoclopramide (%)	Na Alginate (%)	Xylitol (%)	pH	Water (%)
M1	0.8	5	3	4.5	91.2
M2	1.4	3	1	6.0	94.6
M3	0.8	3	1	4.5	95.2
M4	1.4	3	3	4.5	92.6
M5	0.8	3	3	6.0	93.2
M6	**1.1**	**4**	**2**	**5.5**	**92.9**
M7	1.4	5	1	4.5	92.6
M8	0.8	5	1	6.0	93.2
M9	1.4	5	3	6.0	90.6
M10	**1.1**	**4**	**2**	**5.5**	**92.9**
M11	**1.1**	**4**	**2**	**5.5**	**92.9**

**Table 2:** Chromatography gradient elution setting.

Time (min)	Mobile Phase A (Per Cent *V*/*V*)	Mobile Phase B (Per Cent *V*/*V*)
0–1.5	95	5
1.5–16.5	95 → 42.5	5 → 57.5
16.5–17.5	95	5

**Table 3:** Coefficients and its effect on the crystallization of MCP in the film.

MCP	Na-Alginate	Xylitol	pH
11.1	−11.1	−33.5	−6.0

## Data Availability

The data presented in this study are contained within the article. Additional data will be provided by reasonable request.
